# Access to new cancer medicines in Australia: dispelling the myths and informing a public debate

**DOI:** 10.1186/s40545-016-0062-x

**Published:** 2016-04-07

**Authors:** Agnes Vitry, Barbara Mintzes, Wendy Lipworth

**Affiliations:** Faculty of Pharmacy, Bias and Research Integrity Node, Charles Perkins Centre, University of Sydney, Johns Hopkins Drive, Camperdown, NSW 2050 Australia; Centre for Values, Ethics and the Law in Medicine, School of Public Health, Medical Foundation Building K25, The University of Sydney, Sydney, NSW 2006 Australia; School of Pharmacy and Medical Sciences, University of South Australia, GPO Box 2471, Adelaide, SA 5001 Australia

**Keywords:** Cancer medicines, Funding, High prices, Consumer engagement, Transparency, Public debate

## Abstract

Despite the high level of spending on cancer medicines in Australia, consumer organisations and the pharmaceutical industry often make claims of delayed or lack of access to new cancer medicines—claims that are frequently supported by prominent coverage in the Australian media. These claims, while morally and psychologically compelling, tend to ignore the complexity of medicines funding decisions. In this commentary we summarise the current situation regarding the registration and funding of cancer medicines in Australia, elucidate the main challenges associated with access to cancer medicines in the Australian context, and describe some of the steps that have been taken to address these challenges.

## Background

Access to new cancer medicines is currently a highly contentious issue in Australia. Indeed, hardly a week goes by without lack of access to a new ‘breakthrough’ cancer medicine being highlighted in the Australian media. Almost inevitably, existing medicines approval and coverage processes are blamed for the problem. But are current Australian regulatory and funding systems really leading to ‘*second-rate cancer care*’ in Australia as suggested by the Oncology Industry Taskforce (a taskforce of 16 pharmaceutical companies) [1], the Cancer Drugs Alliance (a multi-stakeholder organisation with close ties to the industry) [2] and some lay media? With this question in mind, this commentary summarises the current situation regarding the registration and funding of cancer medicines in Australia, elucidates the main challenges associated with access to cancer medicines in the Australian context, and describes some of the steps that have been taken to address these challenges.

### Registration and funding of new cancer medicines in Australia

Despite a high incidence of cancer, Australia has one of the lowest rates of cancer mortality in the developed world [3]. These positive outcomes are likely due to the implementation of national cancer screening programmes, access to high quality health care services, and universal public financing of effective cancer medicines through the Pharmaceutical Benefits Scheme (PBS) [4]. Consistent with the first objective of the National Medicines Policy, the PBS aims to provide ‘*timely access to the medicines that Australians need, at a cost individuals and the community can afford*’ [5]. In 2013-2014, the Australian government spent AUD$1.5 billion on cancer medicines. This represented one third of the total cost of cancer care and 16 % of total PBS expenditure [6]. Patients have access to these medicines for free in hospitals, or pay a modest co-payment as out-patients ($36.90 for general and $6.00 for concessional beneficiaries for a full-course of chemotherapy treatment).

Although Australia’s invests substantially in cancer medicines, a number of studies have demonstrated either lack of regulatory approval, or delayed approval, of new cancer medicines in Australia compared to similar countries [7, 8]. However, the delay in regulatory approval in Australia has mostly been explained by a delay in pharmaceutical companies’ applications for registration, which were submitted on average 38 weeks later than applications to the US Food and Drugs Administration (FDA) and the European Medicines Agency (EMA) [6]. Another possible contributing factor is that, unlike the FDA and EMA, Australia’s Therapeutic Goods Administration (TGA) does not currently have the capacity to undertake expedited approvals for medicines [9]. In the US, expedited review leads to approvals on average 3.5 months earlier than standard review, but there are serious problems with the FDA’s ability to track and report on post-approval safety following expedited review [10]. Thus the trade-off in expedited review of less complete pre-approval data but more extensive post-market evaluation has failed to fully live up to expectations.

Delayed funding decisions, or lack of funding of new cancer medicines on the PBS, has also been raised as a major concern by some consumer organisations and the pharmaceutical industry [6]. In a study we conducted of PBS approvals of cancer medicines, we found that 44 % (24) of the 56 indications for new cancer medicines approved by the TGA between 2010 and 2013 had also received a positive funding recommendation by the Pharmaceutical Benefit Advisory Committee (PBAC), with an average time between the TGA authorisation and PBAC positive recommendation of 343 days [8]. Similarly, an analysis of 182 submissions for cancer medicines between 2005 and 2014 showed that the overall rate of deferral or rejection was 56 % [11]. The most common reasons for deferring or rejecting listing of new cancer medicines on the PBS were uncertain cost-effectiveness (38 %), unacceptable cost-effectiveness (21 %) and uncertain effectiveness (18 %) [11]. Australia’s PBAC is not alone in these judgments. The proportion of positive funding decisions for cancer medicines between 1994 and 2014 in Australia was similar to rates in the United Kingdom and Canada, two countries which also use economic analyses to inform funding decisions [12].

### Challenges associated with access to cancer medicines in Australia

#### Uncertain and limited benefits of cancer medicines

While the same type of evidentiary standards are applied to the registration and funding of cancer and non-cancer medicines, regulators and payers face particular challenges when it comes to evaluating many cancer medicines. This is largely because the quality of clinical trial evidence on cancer medicines is generally lower than for other therapeutic classes [13, 14]. A retrospective analysis of submissions for cancer medicines considered by the PBAC between 2005 and 2012 found that on average, half of major submissions had significant problems with supporting clinical evidence [15]. Although some new cancer medicines provide important therapeutic benefits, many new cancer medicines, especially those marketed for advanced cancers, fail to lead to gains in survival or lead to only minimal gains over standard care and are sometimes associated with greater toxicity. This makes it very difficult to demonstrate their “value” relative to alternatives [16–19].

#### High prices of cancer medicines

Despite the uncertain evidence of benefit for many new cancer medicines, prices of cancer medicines have grown dramatically in all countries over the past 15 years [20–25]. In Australia, expenditure on chemotherapy has been increasing faster than any other area of health care, with an average annual growth rate of 63 % from 2009-10 to 2013-14 [26]. This increase may be partially explained by a larger number of patients being treated, but is also likely related to the price paid per cancer prescription, which rose by 133 % from AUD$337 in 1999-2000 to AUD$786 in 2011-12—more than double the increase of all other PBS medicines together [27]. A recent survey of the prices of cancer medicines in 16 European countries, Australia and New Zealand showed that Australian prices were generally similar to the median prices of all countries [28]. In industry submissions to PBAC, cancer medicines tend to have a higher cost per quality-adjusted life-year (QALY) than non-cancer medicines: 29 % of cancer medicines versus 15 % of non-cancer medicines have a reported cost per QALY of more than AUD 45,000 [29]. This helps to explain why cancer medicines are less likely to be funded than other therapeutic categories.

### Strategies for improving access to cancer medicines

Australia’s medicines regulation and funding processes are constantly being reviewed and revised in an effort to improve access to safe, effective and cost-effective medicines. In 2014-2015, two national reviews examined policy options for improving medicines regulatory and funding processes in Australia: the Expert Review of Medicines and Medical Devices Regulation [9] and the Australian Senate’s inquiry on ‘*Availability of new, innovative and specialist cancer drugs in Australia*’ [6]. We believe that three issues have emerged as being particularly important, both in reviews and in other contexts: 1) the need to streamline regulatory and funding processes, 2) the need for greater consumer involvement in decision-making and 3) the need to address the problem of high cancer drug prices.

#### Streamlining regulatory and funding processes

A number of steps have recently been taken in Australia to shorten the approval-funding-listing cycle by streamlining administrative procedures. Since January 2011, parallel TGA and PBAC processes have been introduced, thus reducing the time lag between marketing authorisation and funding approval [30]. A single entry point has also been established for speeding applications of medicines with a ‘co-dependent’ diagnostic technology (such as a genetic test for a ‘targeted therapy’) [31]. The two reviews mentioned above also put forward a number of new recommendations to enhance administrative processes. For example, they recommended that Australia should make better use of assessments conducted by comparable overseas regulators, and should expedite assessments in certain circumstances which are yet to be defined.

Another innovative funding pathway that is gaining increasing prominence in Australia and globally is the development of managed entry agreements (MEA). Most MEAs to date in Australia have been financial agreements that involve price or volume rebates, or agreements that link the continuation of funding to evidence of benefit documented at the individual patient level [32]. Managed access programs have been more recently introduced in which continuation of funding is conditional on the subsequent provision of favourable scientific evidence of population-level efficacy. In most cases, the manufacturer would be expected to pay a rebate to the Government should these medicines fail to deliver on their claimed benefits [33]. A few medicines, including four cancer medicines (pilimumab, prembrolizumab and trametinib for advanced melanoma and crizotinib for non-small cell lung cancer), have been recently listed on the PBS as part of managed access programs. However, concerns have been raised about the implementation of these programs in other countries including the quality of the methodology of studies undertaken in ‘real world’ settings, as well as the governance and funding of these programs [34, 35]. It is as yet unclear whether these programs contribute meaningfully to the evaluation of the therapeutic effects of new medicines [36]. Detailed information on MEAs is not publicly available and this lack of transparency is a major drawback because it precludes public understanding of the ways in which decisions about initial and continued funding are made [37, 38]. Furthermore, potential cessation of funding of medicines which are part of MEAs requires ongoing good communication for these decisions to be understood and accepted by the public [39]. Currently, patients who are prescribed medicines with continuation rules are required to sign an informed consent document. A comprehensive risk management plan may be required for medicines which are of part of managed access programs when discontinuation of funding at the national level is an option [39].

#### Increasing consumer engagement in decision-making

The Australian Senate Committee recommended expanding the role of consumers and clinicians in PBAC assessment processes, with the objective of better aligning PBAC’s decisions with stakeholders’ preferences. Increased levels of public and patient involvement in decision-making processes may take several forms including higher number of consumer representatives on decision-making committees, or more robust processes of public consultation. These process are important in contexts where values are likely to conflict. However, they also raise two important issues that need to be addressed if public input is to contribute meaningfully to decision-making. The first is how to manage conflicts of interest, as some patient organisations rely on funding from pharmaceutical companies. Such funding can compromise an organisation’s independence and its ability to solely represent cancer patients’ interests, particularly when PBAC is considering funding of a sponsor’s drug. The second issue is effective management of power imbalances, so that consumers are able to be heard and ultimately contribute to decisions.

Transparency is also important because, although PBAC decisions are not based on a strict utilitarian rationality with a fixed funding threshold, they are often assumed to be so. These assumptions—although incorrect—are able to persist in part because the rationale and the value judgements involved in PBAC decisions are not adequately communicated to the public and patients [39]. This, in turn, is because most of the documentation submitted to the PBAC by the manufacturers and generated during the evaluation process is considered to be commercially confidential, and cannot be released publicly. While Public Summary Documents (PSD), which summarize the evidence basis and the reasons supporting the PBAC decisions have been posted on the Australian Government’s website since 2005 [32], PSDs are highly technical and may be difficult for consumers to understand. Furthermore, sensitive information such as Incremental Cost Effectiveness Ratio, financial implications, proposed prices and details of proposed risk-share arrangements are redacted, and PSDs are released only several months after the PBAC decision has been made. This inadequate communication may lead to misconceptions about the rationale of PBAC’s decisions and misinterpretation of scientific evidence by the patients and the media [6]. However, the pharmaceutical industry opposes greater transparency on the grounds of what it perceives to be ‘legitimate commercial in confidence considerations’ [6].

#### Addressing the high prices of cancer medicines

Although the Australian Senate committee report noted the high cost of cancer medicines, it did not comment on the significant role of pharmaceutical companies in delaying funding decisions by making exaggerated initial price demands to secure the highest prices possible for their products. We believe that this was a significant omission in the report and its recommendations, given that independent experts around the world are now warning that high priced medicines are a major threat to the sustainability of pharmaceutical insurance schemes.

Pharmaceutical companies argue that, in the era of personalised, or targeted, cancer therapy they must charge more in order to recoup the cost of development from a smaller cancer patient group. It is difficult to evaluate these claims because lack of transparency about the costs of drug development makes it impossible to get reliable figures on the real cost of development of new medicines. Industry-affiliated institutions claim that development costs could reach US$2.56 billion per medicine gaining approval [40], but these figures may be grossly overestimated, and others have argued that the development cost is more likely to be around US$125 million per drug [41, 42]. Best estimates of development costs for imatinib, for example, were between US$38 million and US$96 million [43]. By comparison, the global sales for imatinib reached US$4.69 billion or US$390 million per month in 2013 [44]. Moreover, the annual cost of imatinib was US$30,000 per patient per year in 2001 in the US when it received FDA approval, a price set by the company to make it profitable, and then nearly tripled to US$90,000 in 2013, despite imatinib having received additional indications, and being taken by a higher number of patients for longer periods of time than anticipated [22, 45]. In Australia, there was enormous controversy recently when the funding for a new treatment for advanced melanoma, ipulimumab (Yervoy®) was rejected by the PBAC on two occasions because the manufacturer, Bristol Myers Squibb, requested a high price even if this medicine had a low response rate and could cause severe immunological adverse effects [46]. Despite the fact that development of Yervoy® may have cost less than US$400 million [47], Wall Street analysts expected Yervoy® to make US$1.7 billion a year in revenues when approved by FDA in 2011 [47].

In the UK, Professor David Haslam, the chairman of the National Institute for Health and Care Excellence (NICE), which is responsible for appraising the cost-effectiveness of medicines in England, recently declared that ‘*The pharmaceutical industry must be willing to show the public how it prices its drugs—or face losing its trust…If we could have greater transparency about pricing, then a lot of problems could be bypassed—it’s not just that drugs are expensive and NICE says no, it’s about the cost-effectiveness of these medicines’* [48]. In the United States, a group of more than 100 experts in chronic myeloid leukemia (CML), has recently drawn attention to the high prices of cancer drugs [22]. American oncologists have declared that ‘*we cannot continue to accept novel therapeutics with very small benefits for exorbitant prices*’ [49]. In France, independent experts have claimed that ‘*skyrocketing prices for new drugs call for a strong response from citizens, healthcare professionals and health authorities*’ [50]. The World Health Organization Director General Margaret Chan recently decried the astronomical costs of new medicines and asked what was a fair profit for a pharmaceutical company [51].

Given that affordability is such a major issue when it comes to access to high cost cancer medicines, refinements of health technology assessment will, on their own, be insufficient to address the problem of access to cancer medicines in Australia. High prices of cancer medicines are an international problem which require global solutions. Greater transparency of medicine prices and price-setting mechanisms, a greater commitment of funding agencies—particularly US insurers—to value-based pricing schemes, and greater awareness of pricing issues among stakeholders including media, consumer and health professional organisations might all help to promote new business models in which the prices of cancer medicines would reflect actual improvements in health outcomes rather than solely what companies believe the market will bear.

## Conclusions

Australia has implemented rigorous methods to promote access to new medicines, including cancer medicines, while also supporting the equity and the sustainability of its universal pharmaceutical coverage scheme. Decisions about the registration and funding of cancer medicines are often challenging because of insufficient evidence of benefits, and high prices requested by pharmaceutical companies. A number of initiatives may improve and streamline current administrative processes. The development of managed entry programs may also help maintain efficacy and cost-effectiveness standards while meeting public’s expectations but the results of these programs has not yet been evaluated. Better involvement of public and patients in decision-making processes may increase the legitimacy and the acceptability of funding decisions. However, greater transparency concerning the rationale and evidence base supporting reimbursement decisions and the basis for the high prices requested by pharmaceutical companies are still required to inform and enhance public debate.
